# Supplementary material for: Pigment Production by Pseudofusicoccum sp.: Extract Production, Cytotoxicity Activity, and Diketopiperazines Identified
Source: Microorganisms. 2025 Jan 26;13(2):277. doi: 10.3390/microorganisms13020277 (PMC11857561; doi:10.3390/microorganisms13020277)
Supplement: Supplementary file 1 [file microorganisms-13-00277-s001.zip › microorganisms-3304890-supplementary.pdf]

**Figure S1. Blast results for the sequences of the LSU gene that were amplified by PCR. It was used as template in the PCR the genomic the DNA obtained from the fungus analyzed in this study. The amplicons were sequenced using a Sanger methodology.**

select all *100 sequences selected* GenBank Graphics Distance tree of results MSA Viewer

| | Description | Scientific Name | Max Score | Total Score | Query Cover | E value | Per. Ident | Accession |
|---|---|---|---|---|---|---|---|---|
| ☑ | Pseudofusicoccum stromaticum strain CMW 13434 large subunit ribosomal RNA gene, partial sequence | Pseudofusicoccu... | 909 | 909 | 100% | 0.0 | 99.21% | KF766389.1 |
| ☑ | Pseudofusicoccum stromaticum strain CBS 117448 28S large subunit ribosomal RNA gene, partial sequence | Pseudofusicoccu... | 909 | 909 | 100% | 0.0 | 99.21% | DQ377931.1 |
| ☑ | Fusicoccum sp. CBS 117451 28S large subunit ribosomal RNA gene, partial sequence | Fusicoccum sp. C... | 909 | 909 | 100% | 0.0 | 99.21% | DQ377930.1 |
| ☑ | Pseudofusicoccum olivaceum culture CBS:124940 strain CBS 124940 large subunit ribosomal RNA gene, partial sequ... | Pseudofusicoccu... | 904 | 904 | 100% | 0.0 | 99.01% | MH874936.1 |
| ☑ | Pseudofusicoccum stromaticum strain CBS 117449 28S large subunit ribosomal RNA gene, partial sequence | Pseudofusicoccu... | 904 | 904 | 100% | 0.0 | 99.01% | DQ377932.1 |
| ☑ | Pseudofusicoccum ardesiacum CBS 122062 28S rRNA gene, partial sequence; from TYPE material | Pseudofusicoccu... | 898 | 898 | 100% | 0.0 | 98.81% | NG_069902.1 |
| ☑ | Pseudofusicoccum adansoniae CBS 122055 28S rRNA gene, partial sequence; from TYPE material | Pseudofusicoccu... | 898 | 898 | 100% | 0.0 | 98.81% | NG_069901.1 |
| ☑ | Pseudofusicoccum adansoniae voucher MFLUCC 16-0367 28S large subunit ribosomal RNA gene, partial sequence | Pseudofusicoccu... | 898 | 898 | 100% | 0.0 | 98.81% | MH260317.1 |
| ☑ | Pseudofusicoccum olivaceum culture CBS:124941 strain CBS 124941 large subunit ribosomal RNA gene, partial sequ... | Pseudofusicoccu... | 898 | 898 | 100% | 0.0 | 98.81% | MH874937.1 |
| ☑ | Pseudofusicoccum ardesiacum culture CBS:122062 strain CBS 122062 large subunit ribosomal RNA gene, partial seq... | Pseudofusicoccu... | 898 | 898 | 100% | 0.0 | 98.81% | MH874717.1 |
| ☑ | Pseudofusicoccum adansoniae culture CBS:122055 strain CBS 122055 large subunit ribosomal RNA gene, partial seq... | Pseudofusicoccu... | 898 | 898 | 100% | 0.0 | 98.81% | MH874715.1 |
| ☑ | Pseudofusicoccum adansoniae strain MFLUCC 17-0359 large subunit ribosomal RNA gene, partial sequence | Pseudofusicoccu... | 898 | 898 | 100% | 0.0 | 98.81% | MK478925.1 |
| ☑ | Pseudofusicoccum adansoniae strain MFLUCC 17-0339 large subunit ribosomal RNA gene, partial sequence | Pseudofusicoccu... | 898 | 898 | 100% | 0.0 | 98.81% | MK478924.1 |
| ☑ | Pseudofusicoccum adansoniae strain MFLU 19-0242 large subunit ribosomal RNA gene, partial sequence | Pseudofusicoccu... | 898 | 898 | 100% | 0.0 | 98.81% | MK478923.1 |
| ☑ | Pseudofusicoccum adansoniae strain MFLUCC 17-0334 large subunit ribosomal RNA gene, partial sequence | Pseudofusicoccu... | 898 | 898 | 100% | 0.0 | 98.81% | MK478922.1 |
| ☑ | Pseudofusicoccum adansoniae strain MFLUCC 17-0333 large subunit ribosomal RNA gene, partial sequence | Pseudofusicoccu... | 898 | 898 | 100% | 0.0 | 98.81% | MK478921.1 |
| ☑ | Pseudofusicoccum adansoniae strain MFLUCC 17-0327 large subunit ribosomal RNA gene, partial sequence | Pseudofusicoccu... | 898 | 898 | 100% | 0.0 | 98.81% | MK478920.1 |
| ☑ | Pseudofusicoccum ardesiacum strain MFLUCC 17-0323 large subunit ribosomal RNA gene, partial sequence | Pseudofusicoccu... | 898 | 898 | 100% | 0.0 | 98.81% | MK478919.1 |
| ☑ | Pseudofusicoccum ardesiacum Avo16 gene for 28S ribosomal RNA, partial sequence | Pseudofusicoccu... | 898 | 898 | 100% | 0.0 | 98.81% | LC787291.1 |
| ☑ | Pseudofusicoccum ardesiacum Avo4 gene for 28S ribosomal RNA, partial sequence | Pseudofusicoccu... | 898 | 898 | 100% | 0.0 | 98.81% | LC787282.1 |

**Figure S2. Blast results for the sequences of the ITS gene that were amplified by PCR. It was used as template in the PCR the genomic the DNA obtained from the fungus analyzed in this study. The amplicons were sequenced using a Sanger methodology.**

select all *100 sequences selected* GenBank Graphics Distance tree of results MSA Viewer

| | Description | Scientific Name | Max Score | Total Score | Query Cover | E value | Per. Ident | Acc. Len | Accession |
|---|---|---|---|---|---|---|---|---|---|
| ☑ | Fusicoccum sp. NR-2006-D13 18S ribosomal RNA gene, partial sequence; internal transcribed spacer 1 and 5.8S ri… | Fusicoccum sp. … | 874 | 874 | 55% | 0.0 | 98.20% | 548 | DQ480351.1 |
| ☑ | Pseudofusicoccum adansoniae strain C1536 internal transcribed spacer 1, partial sequence; 5.8S ribosomal RNA g… | Pseudofusicoccu… | 854 | 854 | 53% | 0.0 | 98.16% | 566 | KT968485.1 |
| ☑ | Pseudofusicoccum adansoniae strain C1542 internal transcribed spacer 1, partial sequence; 5.8S ribosomal RNA g… | Pseudofusicoccu… | 854 | 854 | 53% | 0.0 | 98.16% | 576 | KT968484.1 |
| ☑ | Pseudofusicoccum adansoniae strain B1683 internal transcribed spacer 1, partial sequence; 5.8S ribosomal RNA g… | Pseudofusicoccu… | 854 | 854 | 53% | 0.0 | 98.16% | 565 | KT968482.1 |
| ☑ | Pseudofusicoccum adansoniae strain B1682 internal transcribed spacer 1, partial sequence; 5.8S ribosomal RNA g… | Pseudofusicoccu… | 854 | 854 | 53% | 0.0 | 98.16% | 564 | KT968481.1 |
| ☑ | Pseudofusicoccum adansoniae strain B1474 internal transcribed spacer 1, partial sequence; 5.8S ribosomal RNA g… | Pseudofusicoccu… | 852 | 852 | 53% | 0.0 | 98.16% | 576 | KT968483.1 |
| ☑ | Pseudofusicoccum sp. isolate PH3 internal transcribed spacer 1, partial sequence; 5.8S ribosomal RNA gene and i… | Pseudofusicoccu… | 850 | 850 | 53% | 0.0 | 98.16% | 572 | MT936883.1 |
| ☑ | Pseudofusicoccum adansoniae strain BT11 internal transcribed spacer 1, partial sequence; 5.8S ribosomal RNA ge… | Pseudofusicoccu… | 848 | 1007 | 53% | 0.0 | 98.15% | 690 | KM357559.1 |
| ☑ | Pseudofusicoccum sp. MT-2008b isolate CMW23076 18S ribosomal RNA gene, partial sequence; internal transcrib… | Pseudofusicoccu… | 841 | 841 | 53% | 0.0 | 98.14% | 520 | EU588624.1 |
| ☑ | Pseudofusicoccum adansoniae strain B0121 18S ribosomal RNA gene, partial sequence; internal transcribed space… | Pseudofusicoccu… | 872 | 872 | 55% | 0.0 | 97.64% | 538 | KJ607148.1 |
| ☑ | Pseudofusicoccum adansoniae strain CMW 26147 18S ribosomal RNA gene, partial sequence; internal transcribed … | Pseudofusicoccu… | 857 | 857 | 55% | 0.0 | 97.60% | 592 | KF766220.1 |
| ☑ | Fungal endophyte isolate MIS01 18S ribosomal RNA gene, partial sequence; internal transcribed spacer 1, 5.8S rib… | fungal endophyte | 857 | 857 | 55% | 0.0 | 97.60% | 529 | JN163854.1 |
| ☑ | Pseudofusicoccum adansoniae isolate MMI00064 18S ribosomal RNA gene, partial sequence; internal transcribed … | Pseudofusicoccu… | 837 | 837 | 53% | 0.0 | 97.55% | 564 | JQ585586.1 |
| ☑ | Pseudofusicoccum adansoniae strain NI320 internal transcribed spacer 1, partial sequence; 5.8S ribosomal RNA g… | Pseudofusicoccu… | 824 | 824 | 53% | 0.0 | 97.52% | 514 | OM462370.1 |
| ☑ | Pseudofusicoccum adansoniae strain AS15 internal transcribed spacer 1, partial sequence; 5.8S ribosomal RNA ge… | Pseudofusicoccu… | 824 | 824 | 53% | 0.0 | 97.52% | 514 | OM462368.1 |
| ☑ | Pseudofusicoccum adansoniae isolate MFLUCC14-0517 internal transcribed spacer 1, partial sequence; 5.8S ribos… | Pseudofusicoccu… | 824 | 905 | 53% | 0.0 | 97.52% | 613 | KM396906.1 |
| ☑ | Pseudofusicoccum adansoniae isolate MFLUCC13-0705 internal transcribed spacer 1, partial sequence; 5.8S ribos… | Pseudofusicoccu… | 824 | 824 | 53% | 0.0 | 97.52% | 523 | KM396904.1 |
| ☑ | Pseudofusicoccum adansoniae strain WAC13295 18S ribosomal RNA gene, partial sequence; internal transcribed s… | Pseudofusicoccu… | 824 | 824 | 53% | 0.0 | 97.52% | 518 | GU172403.1 |
| ☑ | Pseudofusicoccum adansoniae strain WAC13278 18S ribosomal RNA gene, partial sequence; internal transcribed s… | Pseudofusicoccu… | 824 | 824 | 53% | 0.0 | 97.52% | 515 | GU172402.1 |
| ☑ | Pseudofusicoccum adansoniae isolate ZAR-11 small subunit ribosomal RNA gene, partial sequence; internal transc… | Pseudofusicoccu… | 865 | 865 | 55% | 0.0 | 97.45% | 536 | OR073515.1 |

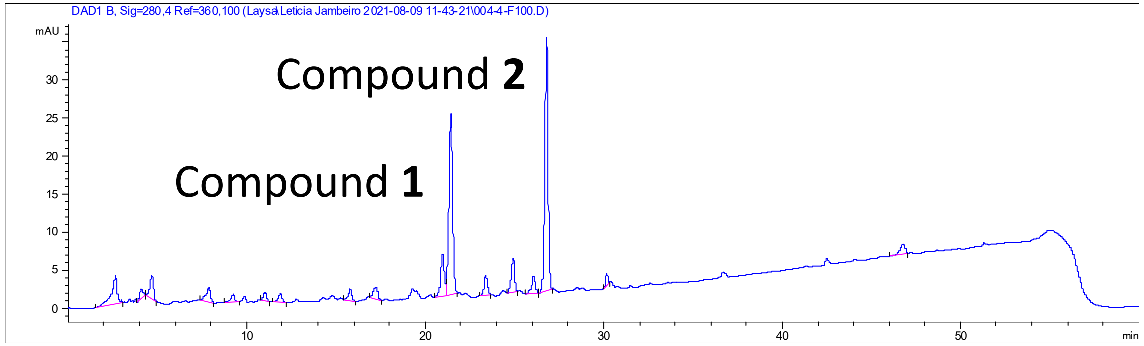


**Figure S3.** Chromatogram of isolation by reverse phase HPLC of the compounds **1** and **2**.

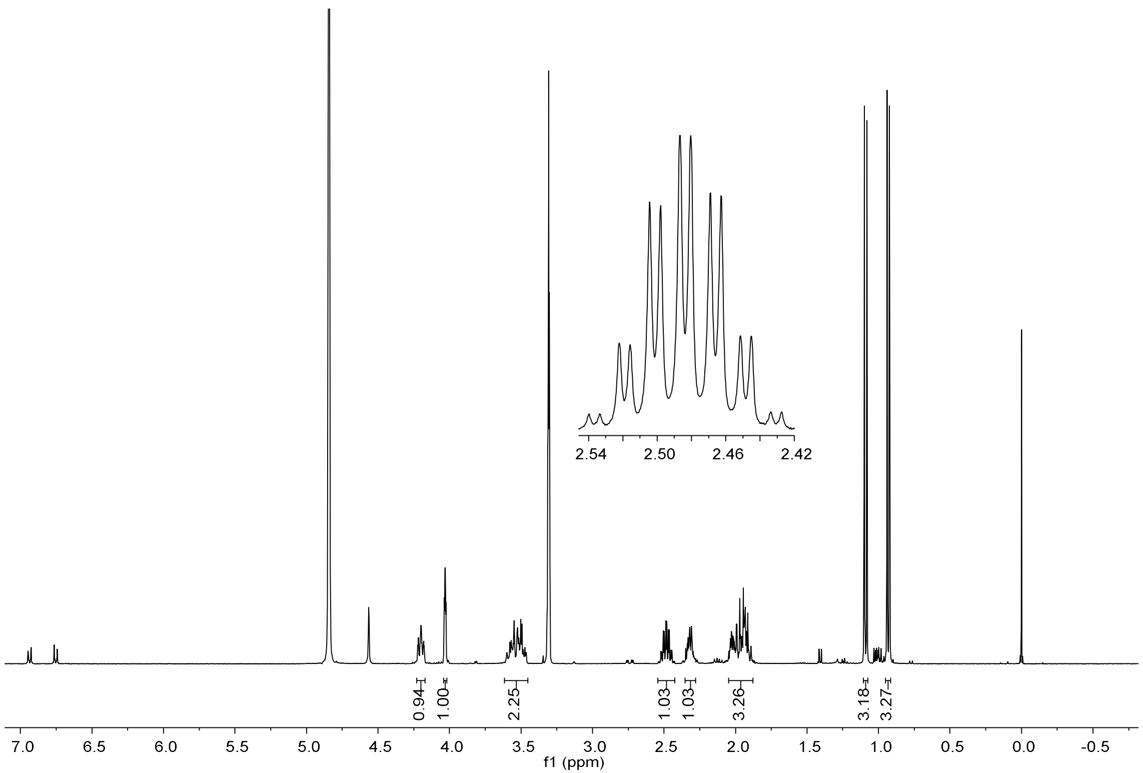


**Figure S4.** NMR spectra of compound **1** (400 MHz $^{1}H$, $CD_3OD$) with expansion.

**Table S1.** NMR data for compound **1** (400 MHz $^1$H, $CD_3OD$) compared with data obtained by Campbell *et al*. in 2009 (300 MHz, $CDCl_3$) and He *et al*., 2013 (500 MHz, $CDCl_3$)

| POSITION | COMPOUND 1<br>$\delta_H$ [ppm] (*J* Hz, H) | CAMPBELL *ET AL*., 2009<br>$\delta_H$ [ppm] (*J* Hz, H) | HE *ET AL*., 2013<br>$\delta_H$ [ppm] |
|---|---|---|---|
| **3** | 4.03 *m* (1H) | 3.94 *s* (1H) | 3.73 |
| **6** | 4.20 *m* (1H) | 4.08 *t* (7,5; 1H) | 4.09 |
| **7, 8a** | 2.04 – 1.88 *m* (3H) | 2.09 – 1.88 *m* (3H) | 1.94 – 2,40, 1.89 |
| **8b** | 2.32 *m* (1H) | 2.39 – 2.33 *m* (1H) | 2.03 |
| **9** | 3.43 – 3,59 *m* (2H) | 3.57 – 3.51 *m* (1H)<br>3.67 – 3.60 *m* (1H) | 3.52<br>3.69 |
| **10** | 2.48 *m* (1H) | 2.63 *sep of d* (6.9, 2.7, 1H) | 2.24 |
| **11** | 0.93 *d* (6.9, 3H) | 0,92 *d* (6.9; 3H) | 0.99 |
| **12** | 1.09 *d* (6.9, 3H) | 1,09 *d* (6.9, 3H) | 1.04 |

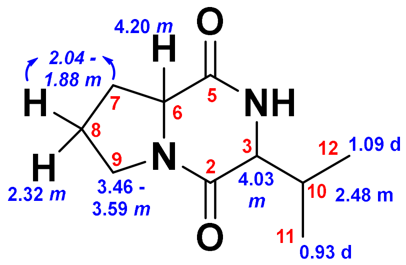


**Figure S5.** Proposed structure and [1]H assignment of compound **1**: *cyclo*(L-Pro-L-Val) $C_{10}H_{16}N_2O_2$

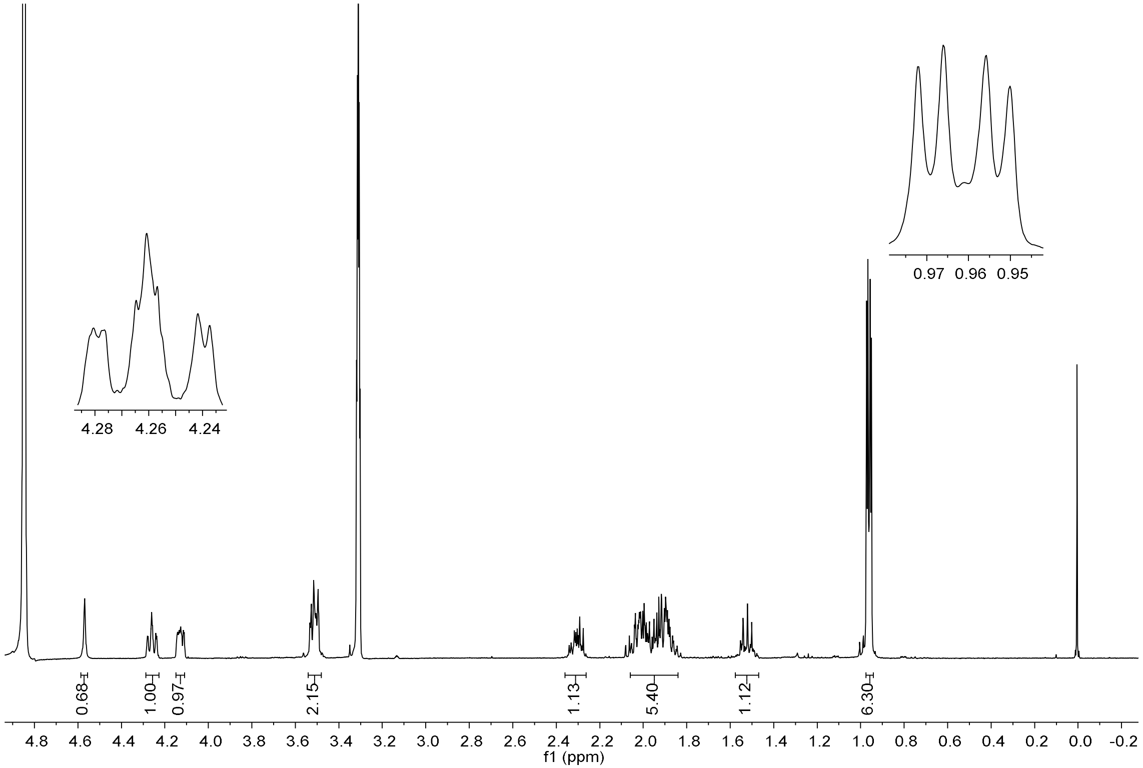


**Figure S6.** NMR spectra of compound **2** (400 MHz $^{1}$H, $CD_3OD$) with expansions.

**Table S2.** NMR data for compound **2** (400 MHz $^{1}H$, $CD_3OD$) compared with data obtained by Campbell *et al*. in 2009 (300 MHz, $CDCl_3$) and He *et al*., 2013 (500 MHz, $CDCl_3$).

<table>
<tr><th rowspan="2">POSITION</th><th>COMPOUND 2</th><th>CAMPBELL ET AL., 2009</th><th>HE ET AL., 2013</th></tr>
<tr><th>$\delta_H$ [ppm] (J Hz, H)</th><th>$\delta_H$ [ppm] (J Hz, H)</th><th>$\delta_H$ [ppm]</th></tr>
<tr><td>3</td><td>4.14 – 4.11 m (1H)</td><td>4.11 t (7.9, 1H)</td><td>4.10</td></tr>
<tr><td>4</td><td>4.56 s (1H)</td><td>6.22 br s (1H)</td><td>6.18</td></tr>
<tr><td>6</td><td>4.26 ddd (1.7, 7.1, 8.6, 1H)</td><td>4,00 dd (1H)</td><td>4.00</td></tr>
<tr><td>7,10,11</td><td>2.06 – 1.84 m (5H)</td><td rowspan="2">2.19 – 1.77 m (6H)</td><td>2.10 – 2.33, 1.51-2.05, 1.77</td></tr>
<tr><td>8a</td><td>1.55 – 1.50 m (1H)</td><td>1.88</td></tr>
<tr><td>8b</td><td>2.33 – 2.27 m (1H)</td><td>2.38 – 2.30 m (1H)</td><td>2.01</td></tr>
<tr><td>9</td><td>3.53 – 3.49 m (2H)</td><td>3.62 – 3.49 m (2H)</td><td>3.52 – 3.59</td></tr>
<tr><td>12</td><td>0.95 d (6.9, 3H)</td><td>0.95 d (6.9, 3H)</td><td>0.98</td></tr>
<tr><td>13</td><td>0.97 d (6.9, 3H)</td><td>0.99 d (6.3, 3H)</td><td>0.93</td></tr>
</table>

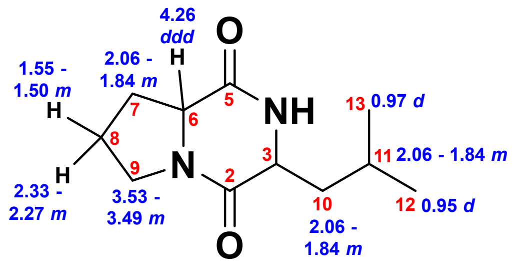


**Figure S7.** Proposed structure and [1]H assignment of compound **2**: *cyclo*(L-Leu-L-Pro) $C_{11}H_{18}N_2O_2$
